# Design Optimization of Double-Gate Isosceles Trapezoid Tunnel Field-Effect Transistor (DGIT-TFET)

**DOI:** 10.3390/mi10040229

**Published:** 2019-03-30

**Authors:** Hwa Young Gu, Sangwan Kim

**Affiliations:** Department of Electrical and Computer Engineering, Ajou University, Suwon 16499, Korea; dogn1006@ajou.ac.kr

**Keywords:** tunnel field-effect transistors (TFETs), ambipolar current, scaling, subthreshold swing, FinFET

## Abstract

Recently, tunnel field-effect transistors (TFETs) have been regarded as next-generation ultra-low-power semi-conductor devices. To commercialize the TFETs, however, it is necessary to improve an on-state current caused by tunnel-junction resistance and to suppress a leakage current from ambipolar current (*I*_AMB_). In this paper, we suggest a novel TFET which features double gate, vertical, and trapezoid isosceles channel structure to solve the above-mentioned technical issues. The device design is optimized by examining its electrical characteristics with the help of technology computer-aided design (TCAD) simulation. As a result, double-gate isosceles trapezoid (DGIT) TFET shows a much better performance than the conventional TFET in terms of ON-state current (*I*_ON_), *I*_AMB_, and gate-to-drain capacitance (*C*_GD_). It is confirmed that an inverter composed of DGIT-TFETs can operate with less than 1 ns intrinsic delay time and negligible voltage overshoot.

## 1. Introduction

Over the past several decades, complementary metal-oxide-semi-conductor (CMOS) technologies have been scaled down to improve integration densities and performance [1,2,3]. As the integration density increases, however, the increase of power consumption becomes an emerging main concern. Since the power dissipation is proportional to the square of supply voltage (*V*_DD_), future CMOS devices should be operating with low *V*_DD_. However, MOS field-effect transistors (MOSFETs) have a limit of 60 mV/dec subthreshold swing (*S*) at room temperature because they are based on a thermionic carrier injection. As a result, it is fundamentally impossible to lower *V*_DD_ maintaining a high on-off current ratio (*I*_ON_/*I*_OFF_) [4]. Therefore, a sharp-switching device, based on a novel operating mechanism, is needed to achieve sub-60 mV/dec-*S*, and hence ultra-low power operation. Recently, tunnel FETs (TFETs) have been extensively investigated as one of the promising candidates for a next-generation low-power logic element [5,6,7,8,9,10,11]. Because TFETs inject charges through a band-to-band tunneling (BTBT) mechanism from a source to a channel, abrupt switching is possible compared to conventional MOSFETs with drastically reduced *I*_OFF_ [12,13,14]. In addition, they are able to inherit MOSFETs technologies with minimum cost and maximum efficiency with the help of similar structure and process to MOSFETs used in current CMOS technologies [15].

However, TFETs have some technical challenges to be solved for succeeding or alternating MOSFETs. First, they suffer from a low-level *I*_ON_ and a worse *S* than expectation due to a high tunnel resistance [16]. A multi-gate structure and a narrow bandgap material (e.g., SiGe or Ge) are regarded as promising strategies to address the above-mentioned issues by improving gate controllability and BTBT efficiency [16,17,18]. In addition, heterojunction is preferred to suppress the *I*_OFF_ caused by Shockley-Read-Hole (SRH) recombination, which is exponentially increased in narrow band-gap materials [19]. However, in case of a conventional lateral-channel structure, there is a process capability issue for forming SiGe-Si heterojunction [20], with abrupt doping profile aligning with gate.

The second technical challenge is ambipolar current (*I*_AMB_), which is attributed to the BTBT at the channel-to-drain junction and causes a conduction of current during both positive and negative gate voltages (*V*_GS_) [21]. Lowering a drain doping concentration (*N_D_*) and introducing an underlap between gate and drain have been studied to address it [22,23]. Because TFETs have low-level driving currents, the effect of increasing resistance (e.g., drain resistance and contact resistance), due to a lightly doped drain, is negligible. However, if the driving current of TFETs is eventually improved, it will not be an ultimate solution because it will act as a new bottleneck in current drivability [24]. Similarly, the length of drain underlap region (DU) should be minimized since it increases parasitic resistance and degrades integration density.

Therefore, in this paper, a new structure TFET is proposed to address the abovementioned issues (i.e., *I*_ON_, *I*_OFF_, and *S*), simultaneously. In addition, its electrical characteristics are analyzed and optimized using technology computer-aided design (TCAD) simulation [25]. This paper is organized as follows: In Section 2, the key features of device design, and the parameters used in TCAD, simulation are described. In Section 3, the device design is optimized in terms of direct current (DC) and alternating current (AC) characteristics, depending on the several design parameters. Finally, the results are summarized and concluded in Section 4.

## 2. Double-Gate Isosceles Trapezoid TFET (DGIT-TFET)

**Figure 1:**
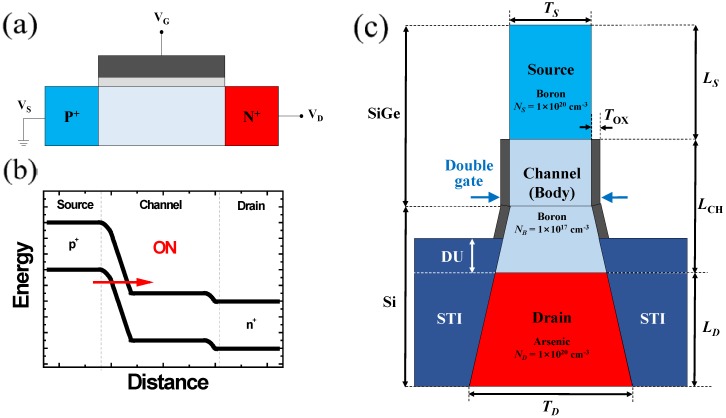
(**a**) A cross-sectional schematic and (**b**) an energy band diagram at on-state of conventional *n*-channel tunnel field-effect transistor (TFET). When positive *V*_GS_ is applied, carrier injection through BTBT mechanism occurs from the source to the channel. (**c**) A cross-sectional schematic of *n*-channel DGIT-TFET. Definitions of abbreviations are summarized in Table 1.

Figure 1 shows a structure of double-gate isosceles trapezoid TFET (DGIT-TFET) studied in this work. It adopts a double-gate (DG) structure to enhance gate controllability over the channel. It features a vertical channel structure, in which the source and drain are located at a narrow top, and relatively thick bottom regions, respectively. The vertical structure is advantageous, not only for increasing the integration density without any areal penalty, but also for adopting a selective epitaxial layer growth (SEG) technique to improve *I*_ON_/*I*_OFF_ with the help of heterojunction [26]. In this study, the Si_1-x_Ge_x_-channel is overlapped with the gate by 15 nm considering the process margin in SEG process (Figure 1). It is also helpful to improve the *I*_ON_ further by using pseudo-direct BTBT when the Ge mole fraction is increased [16,18,27]. The channel length (*L*_CH_) is set by 30 nm to exclude short-channel effects and equivalent gate oxide thickness (*T*_OX_) is set by 0.5 nm assuming high-k dielectric. The other important design parameters are summarized in Table 1, unless otherwise noted [28]. The electrical characteristics of DGIT-TFET, depending on the design parameters are investigated, and analyzed using Synopsys Sentaurus^TM^ (Synopsys, Mountain View, CA, USA) [25]. For a rigorous examination, Shockley-Read-Hall (SRH) and dynamic non-local BTBT models are used after calibration. In detail, *A* and *B* parameters in Kane’s model is changed as in [18], to consider both indirect and direct BTBT components, simultaneously. The modified local density approximation (MLDA) model is also used for the consideration of quantum effect.

**Figure 2:**
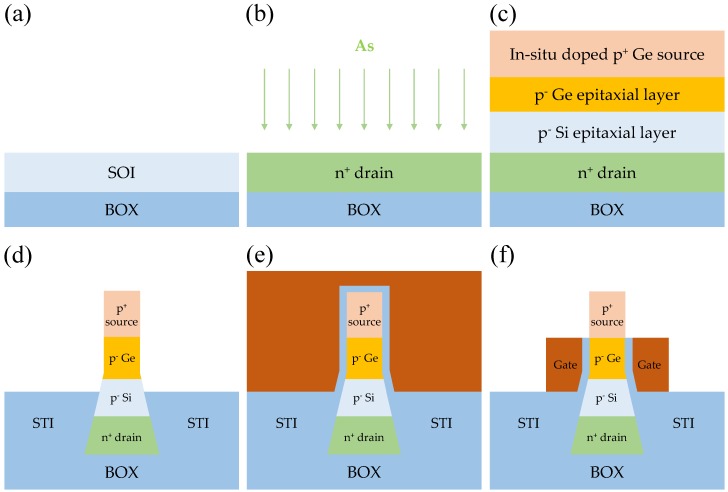
An exemplary process flow for an *n*-channel DGIT-TFET. (**a**) Either silicon-on-insulator (SOI) or bulk-Si wafer can be used as a substrate. (**b**) N-type drain formation with As^+^ ion implantation. (**c**) Channel and source regions can be formed by in-situ doped epitaxial layer growth technique. (**d**) Formation of tapered structure with the help of conventional shallow trench isolation (STI) processes. (**e**,**f**) Gate stack formation with high-k/metal gate.

The *n*-channel DGIT-TFET can be fabricated by the process flow, shown in Figure 2. Starting with a silicon-on-insulator (SOI) wafer (a) drain region is formed by arsenic (As) ion implantation (b). A bulk-Si substrate can alternate the SOI with the help of vertical structure of DGIT-TFET. The sequential in-situ, doped epitaxial growths are performed for channel (i.e., lightly doped p^−^ Si and Ge layers) and source (i.e., highly doped p^+^ Ge layer) (c). After patterning tapered structure, conventional shallow trench isolation (STI) process is performed by oxide gap-fill, chemical mechanical polishing (CMP), and STI wet-etching processes in sequence (d). The length of DU can simply be adjusted by changing STI-oxide wet-etching time. After dopant activation, atomic layer deposition (ALD) for high-k gate oxide is followed by metal gate deposition (e). Finally, double-gates are formed by side-wall spacer technique, with an appropriate over-etching, to avoid gate-to-source overlap (f). The back-end-of-line (BEOL) processes are not shown here, since the conventional techniques are applicable.

**Figure 3:**
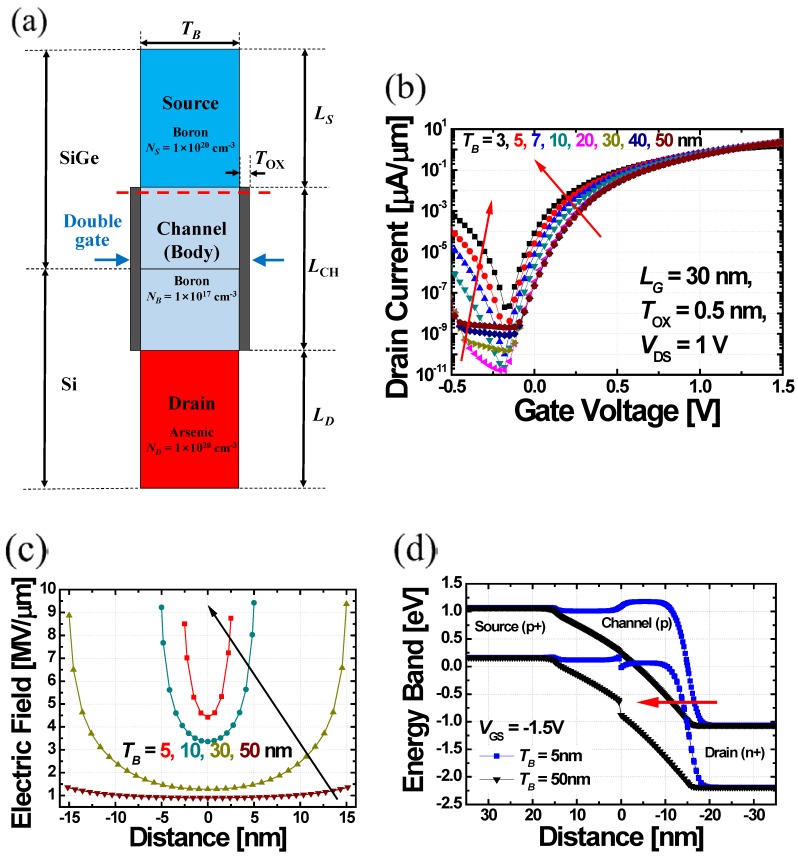
(**a**) The cross-sectional schematic of DG-TFET (**b**) and its current-voltage (*IV*) characteristics, depending on the *T_B_* from 3 to 50 nm. (**c**) Extracted electric field at source-to-channel side (i.e., a red dash line in (a) when *T_B_* is 5, 10, 30, and 50 nm. The increase of electric field, with the smaller *T_B_*, is a clear evidence that the gate controllability over the channel increases (*V*_GS_ = 1.5 V and *V*_DS_ = 1.0 V). (**d**) Energy band diagrams from source to drain in the cases of *T_B_* = 5 (blue) and 50 nm (black) (*V*_GS_ = −1.5 V and *V*_DS_ = 1.0 V). Similar to the reason of *I*_ON_ increase in (b), holes in drain conduction band can be injected into channel with higher BTBT probability as *W*_TUN_ becomes smaller with the thinner *T_B_*.

In order to estimate the effect of asymmetric body thickness (*T_B_*) in DGIT-TFET (i.e., thin source and thick drain) on its electrical characteristics, drain current (*I_D_*) as a function of *V*_GS_ with different *T_B_* are examined in the conventional DG-TFET structure (Figure 3a). The simulation results show that the *I*_ON_ and *S* are improved as *T_B_* becomes thinner (Figure 3b). It is attributed to the improved gate controllability over the channel, which is confirmed by the increase in electric field at source-to-channel junction as *T_B_* decreases (Figure 3c). Unfortunately, there is a drawback that the *I*_AMB_ is also increased with the thinner *T_B_* since tunnel barrier width (*W*_TUN_) at channel-to-drain junction is decreased as well (Figure 3d). On the other hand, it is expected that the DGIT-TFET’s asymmetric source/drain thicknesses will allow it to achieve high *I*_ON_ and low *I*_OFF_, simultaneously.

## 3. Design Optimization of DGIT-TFET

**Figure 4:**
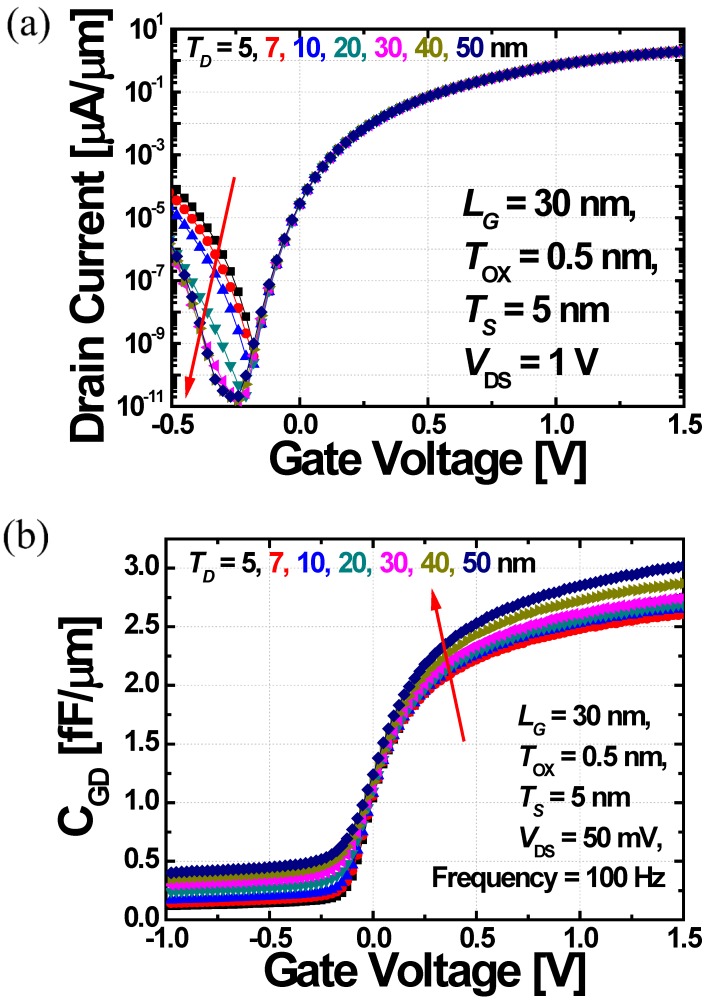
DGIT-TFET’s (**a**) *I_D_* and (**b**) *C*_GD_ as a function of *V*_GS_ while the *T_D_* changes from 5 to 50 nm.

Figure 4a shows the transfer characteristics of DGIT-TFET by changing the drain thickness (*T_D_*) from 5 to 50 nm, while the source thickness (*T_S_*) is fixed at 5 nm considering process capability and compatibility with sub-7 nm technology node [29]. In case of 5 nm-thick *T_D_*, DGIT-TFET is identical to the conventional DG-TFET in Figure 3a,b which shows improved *I*_ON_ but suffers from *I*_AMB_. On the other hand, it is clear that DGIT-TFET can suppress *I*_AMB_, without any *I*_ON_ and *S* degradation, by increasing *T_D_* (Figure 4a). The simulation result shows that *I*_AMB_ is reduced approximated 2 orders of magnitude as *T_D_* increases from 5 nm to 20 nm, since the electric field at the channel-to-drain junction is decreased efficiently.

**Figure 5:**
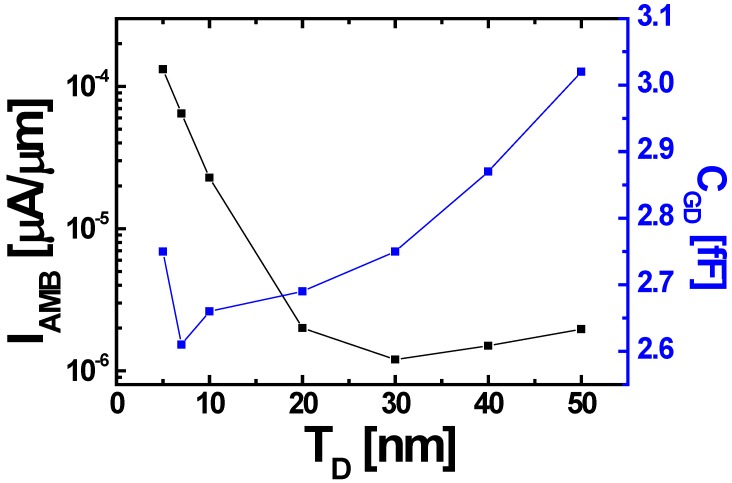
*I*_AMB_ and *C*_GD_ depending on *T_D_* from 5 to 50 nm.

In addition to the effects of *T_D_* on the DC characteristics, the influences of *T_D_* on the AC performances are examined as well. In case of TFET, unlike to the MOSFET, gate-to-drain capacitance (*C*_GD_) dominates entire gate capacitance (*C*_GG_) while gate-to-source capacitance (*C*_GS_) is negligible [30]. Therefore, *C*_GD_ as a function of *V*_GS_, is examined with the various *T_D_* from 5 to 50 nm-thick. Figure 4b shows that *C*_GD_ is increased proportionally to the *T_D_*, due to the increase of drain area. It is problematic for high-speed and low-power CMOS logic applications, since the *C*_GD_ is directly related to the Miller capacitance, which increases voltage over/under-shoots and delay time [31]. In other words, there is a trade-off between *I*_AMB_ and *C*_GD_ in terms of *T_D_*. As shown in Figure 5, the *C*_GD_ remarkably increases when *T_D_* ≥ 20 nm while the amount of decreasing *I*_AMB_ is negligible. Therefore, the optimum *T_D_* is determined as 20 nm.

**Figure 6:**
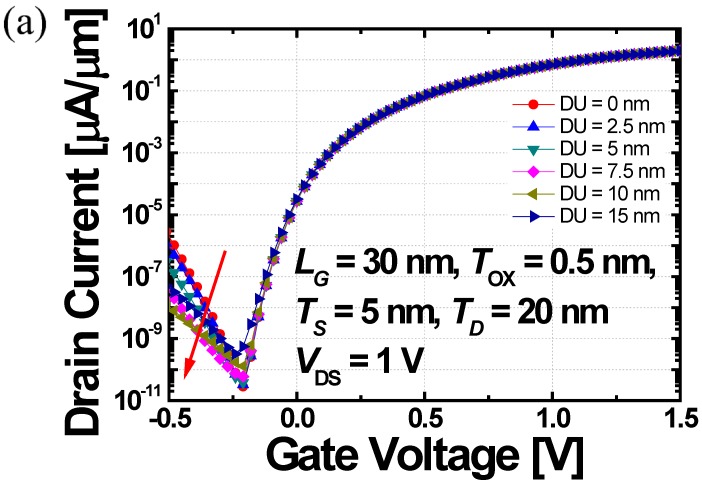

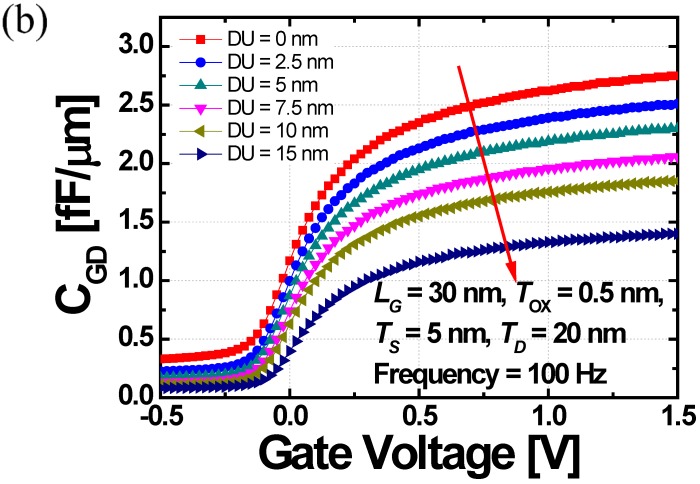
(**a**) *I_D_* and (**b**) *C*_GD_ curves of DGIT-TFET as a function of *V*_GS_ while the DU changes from 0 to 15 nm.

In addition to the increase in *T_D_*, another strategy is required to suppress *I*_AMB_ and *C*_GD_, simultaneously. As shown in Figure 6a, if the DU (i.e., the length of drain underlap region) is increased, the *I*_AMB_ is further decreased. This result is obvious based on the previous studies [32,33]. However, DGIT-TFET can minimize the DU because *I*_AMB_ is already restrained by large *T_D_*. It is beneficial, not only for the small parasitic resistance, but for the high integration density. Moreover, Figure 6a clearly shows that if the DU increases more than 10 nm, the *I*_OFF_ becomes worse in spite of the longer DU due to the significant SRH leakage. The DGIT-TFET with 10 nm-DU shows smaller *I*_AMB_ and *C*_GD_ than that for 0 nm-DU with the amount of about 2.1, and 3.5 orders of magnitudes, respectively (Figure 6a,b). Considering these results, the optimum DU can be determined as ~10 nm. The adoption of drain underlap region can be realized easily without any aggressive process capability issue by changing the height of STI oxide. The detail about the influence of *C*_GD_ on voltage overshoot during CMOS operation will be discussed at the end of this section.

**Figure 7:**
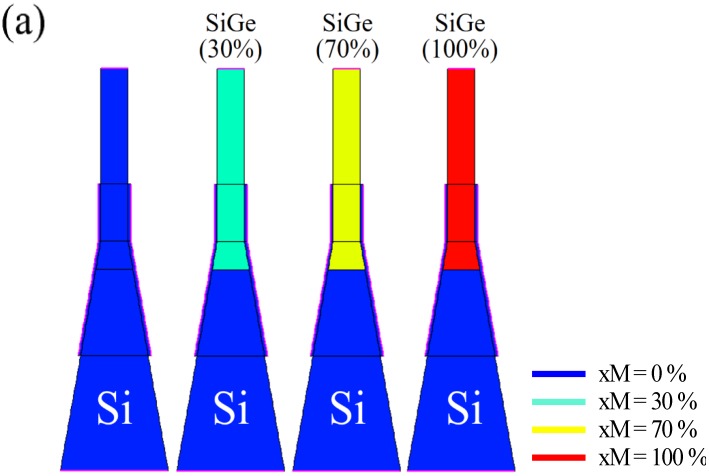

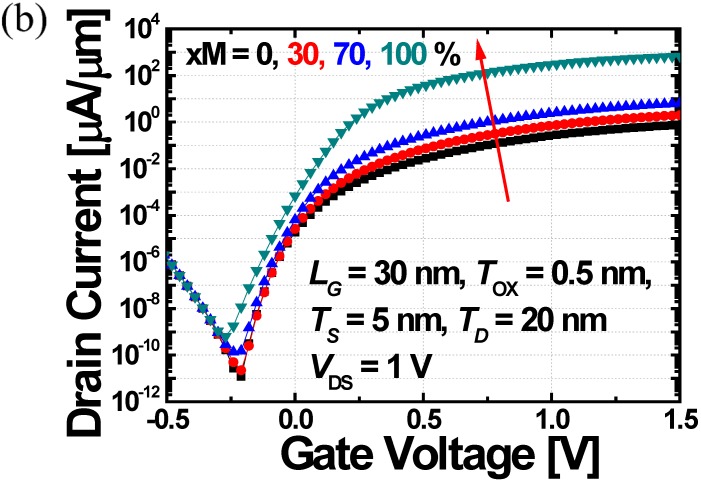
(**a**) The structures of heterojunction Si_1−x_Ge_x_ according to changing Ge mole fraction from 0 to 100 % on source and source-side channel. (**b**) *I_D_* as a function of *V*_GS_ for the structures in (a). As the Ge mole fraction of Si_1−x_Ge_x_ is higher, the *I*_ON_ level is accordingly higher.

As above-mentioned, the vertical-structured DGIT-TFET is compatible to the SEG process for Si_1−x_Ge_x_/Si heterojunction formation. It is worthwhile to study the effects of heterojunction on DGIT-TFET’s driving current, since the use of a narrow bandgap material can reduce the tunnel resistance drastically. Figure 7b shows transfer characteristics of DGIT-TFET according to the Ge mole fraction (xM) at source-channel junction (Figure 7a). If xM increases, *I*_ON_ is effectively improved, without increasing *I*_AMB_, due to the decrease of BTBT resistance. In case of 100%-xM, *I*_ON_ is increased more than two-orders of magnitude from that for sub-70%-xM cases because direct band-to-band tunneling (BTBT) can be utilized [16,18,27].

**Figure 8:**
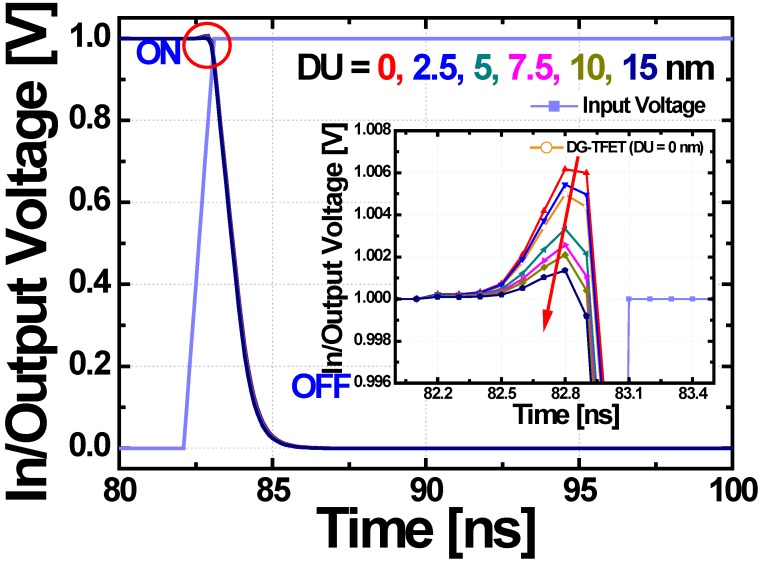
Transient responses of CMOS inverter composed of *n*-channel DGIT-TFET and *p*-channel DGIT-MOSFET during the input signal rising. The inset shows the voltage overshoots can be efficiently suppressed as DU increase. The graph with open symbols also compares in case the conventional structure without tapering (i.e., DG-TFET) is used as a pull-down device. The overshoot trends are exactly matched with *C*_GD_ characteristics shown in Figure 4b and Figure 6b.

Last of all, the transient characteristics of CMOS inverter composed of *n*-channel DGIT-TFET and *p*-channel DGIT-MOSFET are investigated by changing DU. In this case, 100%-xM is used as a source-channel junction for best performance. As shown in Figure 8, it is clear that DGIT-TFET inverter can be operated with less than 1 ns intrinsic delay time. There is a considerable voltage overshoot for the 0 nm-DU due to the large Miller capacitance; *C*_GD_. It is necessary to address this issue since it is problematic in terms of power consumption, reliability, and so on. As shown in the inset of Figure 8, the overshoot phenomenon is significantly suppressed as DU increases with the help of decreased *C*_GD_ (Figure 6b). If 10 nm-DU (the optimized length considering *I*_OFF_ and *C*_GD_) is adopted in DGIT-TFET, overshoot voltage becomes ~30 % of that for 0 nm-DU.

## 4. Summary

In this paper, a novel vertical-channel DG TFET, with asymmetric source/drain area, has been proposed and optimized by using TCAD simulations. It can achieve improved DC, as well as AC performances (i.e., improved *I*_ON_, suppressed *I*_AMB_ and *C*_GD_), with the help of its geometrical benefits. Since the proposed structure is compatible with the SEG process, its performance can be further improved by adopting Si_1-x_Ge_x_ heterojunction at source-channel junction, with high xM. In addition, its high compatibility with state-of-the-art FinFET process flow promises its feasibility of a readily introduction to the current CMOS technology as a successor and/or supplementary for MOSFETs.

## Figures and Tables

**Table 1:** Parameters of double-gate isosceles trapezoid (DGIT-TFET) using technology computer-aided design (TCAD) simulation.

Abbreviations	Definitions	Values
*N_S_*	source doping concentration	Boron, 1 × 10^20^ cm^−3^
*N*_B_	channel doping concentration	Boron, 1 × 10^17^ cm^−3^
*N_D_*	drain doping concentration	Arsenic, 1 × 10^20^ cm^−3^
*L*_CH_	channel length	30 nm
*L_S_ = L_D_*	charge neutral region length	20 nm
*T*_OX_	equivalent gate oxide thickness	0.5 nm
*T_S_*	source region thickness	5 nm
*T_D_*	drain region thickness	20 nm
*V*_DS_	drain voltage	1 V
$\phi_{m}$	gate work function	4.0 eV

## References

[1] International Roadmap for Devices and Systems 2016. https://irds.ieee.org/reports.

[2] Haron N.Z., Hamdioui S. Why is CMOS scaling coming to an END?. Proceedings of the IEEE 3rd International Design and Test Workshop.

[3] BintiMdSallah S.S., Mohamed H., Mamun M., Amin M.S. (2012). CMOS downsizing: Present, past and future. J. Appl. Sci. Res..

[4] Adrian M. (2011). Ionescu and Heike Riel. Tunnel field-effect transistors as energy-efficient electronic switches. Nature.

[5] Seabaugh A.C., Zhang Q. (2010). Low-voltage tunnel transistors for beyond CMOS logic. Proc. IEEE.

[6] Boucart K., Ionescu A.M. (2007). Double-gate tunnel FET with high-k gate dielectric. IEEE Trans. Electron Devices.

[7] Lu H., Seabaugh A. (2014). Tunnel field-effect transistors: State-of-the-art. IEEE J. Electron Devices Soc..

[8] Avci U.E., Morris D.H., Hasan S., Kotlyar R., Kim R., Rios R., Nikonov D.E., Young I.A. Energy efficiency comparison of nanowire heterojunction TFET and Si MOSFET at L_g_ = 13 nm, including P-TFET and variation considerations. Proceedings of the IEEE International Electron Devices Meeting (IEDM).

[9] Robbins M.C., Koester S.J. Crystal-oriented black phosphorus TFETs with strong band-to-band-tunneling anisotropy and subthreshold slope nearing the thermionic limit. Proceedings of the IEEE International Electron Devices Meeting (IEDM).

[10] Kwon H.T., Kim S.W., Lee W.J., Wee D.H., Kim Y. (2016). A recessed-channel tunnel field-effect transistor (RTFET) with the asymmetric source and drain. JSTS.

[11] Kim S.W., Kim J.H., Liu T.-J.K., Choi W.Y., Park By. (2015). Demonstration of L-shaped Tunnel Field-Effect Transistors. IEEE Trans. Electron Devices.

[12] Shih P.-C., Huang H.-C., Wang C.-A., Li J.-Y. A novel vertical tunnel FET of band-to-band tunneling aligned with gate electric field with averaged SS of 28 mV/decade. Proceedings of the Silicon Nanoelectronics Workshop (SNW).

[13] Cristoloveanu S., Wan J., Zaslavsky A. (2016). A review of sharp-switching devices for ultra-low power applications. IEEE J. Electron Devices Soc..

[14] Choi W.Y., Park B.G., Lee J.D. (2007). Tunneling Field-Effect Transistor (TFETs) With Subthreshold Swing (SS) Less Than 60 mV/dec. IEEE Electron Device Lett..

[15] Wu Y.-T., Chiang M.-H., Chen J.F., Ding F., Connelly D., Liu T.-J.K. High-Density SRAM Voltage Scaling Enabled by Inserted-Oxide FinFET Technology. Proceedings of the IEEE SOI-3D-Subthreshold Microelectronics Technology Unified Conference (S3S).

[16] Krishnamohan T., Kim D., Raghunathan S., Saraswat K. Double-gate strained-Ge heterostructure tunneling FET (TFET) with record high drive currents and <60 mV/dec subthreshold slope. Proceedings of the IEEE International Electron Devices Meeting (IEDM).

[17] Kuhn K.J., Murthy A., Kotlyar R., Kuhn M. (2010). Past, Present and Future: SiGe and CMOS Transistor Scaling. ECS Trans..

[18] Kao K.-H., Verhulst A.S., Vandenberghe W.G., Soree B., Groeseneken G., de Meyer K. (2012). Direct and Indirect Band-to-Band Tunneling in Germanium-Based TFETs. IEEE Trans. Electron Devices.

[19] Schenk A., Sant S., Moselund K., Riel H. The impact of hetero-junction and oxide-interface traps on the performance of InAs/Si and InAs/GaAsSb nanowire tunnel FETs. Proceedings of the International Conference on Simulation of Semiconductor Processes and Devices (SISPAD).

[20] Kunii Y., Inokuchi E.Y. (2002). Vertical SiGe epitaxial growth system. Hitachi Rev..

[21] Narang R., Saxena M., Gupta R.S., Gupta M. (2012). Assessment of ambipolar behavior of a tunnel FET and influence of structural modifications. J. Semicond. Technol. Sci. JSTS.

[22] Abdi D.B., Kumar M.J. (2014). Controlling Ambipolar Current in Tunneling FETs Using Overlapping Gate-on-Drain. IEEE J. Electron Devices Soc..

[23] Saurabh S., Kumar M.J. (2016). Fundamentals of Tunnel Field-Effect Transistors.

[24] Kwon D.W., Kim J.H., Park B.-G. (2016). Effects of drain doping concentration on switching characteristics of tunnel field-effect transistor inverters. Jpn. J. Appl. Phys..

[25] Sentaurus^TM^ Device User Guide, ver. K-2015.06, Synopsys Inc.. http://www.sentaurus.dsod.pl/manuals/data/sdevice_ug.pdf.

[26] Pala M.G., Brocard S. (2015). Exploiting Hetero-Junctions to Improve the Performance of III–V Nanowire Tunnel-FETs. IEEE Electron Devices Soc..

[27] Kim S.W., Choi W.Y. (2016). Hump Effects of Germanium/Silicon Heterojunction Tunnel Field-Effect Transistors. IEEE Trans. Electron Devices.

[28] Choi W.Y., Song J.Y., Lee J.D., Park Y.J., Park B.-G. (2005). A novel biasing scheme for I-MOS (impact-ionization MOS) devices. IEEE Trans. Nanotechnol..

[29] Sicard E. (2017). Introducing 7-nm FinFET technology in Microwind. Arch. Ouvert. HAL.

[30] Yang Y., Tong X., Yang L.-T., Guo P.-F., Fan L., Yeo Y.-C. (2010). Tunneling field-effect transistor: Capacitance components and modeling. IEEE Electron Device Lett..

[31] Kwon D.W., Kim J.H., Park E., Lee J., Kim S., Park B.-G. (2017). Switching Characteristics Analysis of Tunnel Field-Effect Transistor (TFET) Inverters. J. Nanosci. Nanotechnol..

[32] Vandenberghe W.G., Verhulst A.S. (2012). Tunnel Field-Effect Transistor with Gated Tunnel Barrier. U.S. Patent.

[33] Verhulst A.S., Vandenberghe W.G., Maex K., Groeseneken G. (2007). Tunnel field-effect transistor without gate-drain overlap. Appl. Phys. Lett..

